# Outpatient hospital attendances in people with rheumatoid arthritis during the COVID-19 pandemic and beyond: a cohort study in three nations of the UK

**DOI:** 10.1093/rheumatology/keaf559

**Published:** 2025-10-24

**Authors:** Ruth E Costello, Michael Parker, Jonathan Kennedy, Sinead Brophy, Amir Mehrkar, Sebastian Bacon, Ben Goldacre, Brian MacKenna, Dave Evans, Laurie A Tomlinson, Rosemary J Hollick, Jenny H Humphreys, The OpenSAFELY Collective, The RHEUMAPS Study Investigators and the LH&W NCS (or CONVALESCENCE) Collaborative, Alex J Walker, Brian MacKenna, Peter Inglesby, Ben Goldacre, Helen J Curtis, Caroline E Morton, Jessica Morley, Amir Mehrkar, Sebastian C J Bacon, George Hickman, Richard Croker, David Evans, Tom Ward, Nicholas J DeVito, Louis Fisher, Amelia C A Green, Jon Massey, Rebecca M Smith, William J Hulme, Simon Davy, Colm D Andrews, Lisa E M Hopcroft, Henry Drysdale, Iain Dillingham, Robin Y Park, Rose Higgins, Christine Cunningham, Milan Wiedemann, Linda Nab, Steven Maude, Orla Macdonald, Ben F C Butler-Cole, Thomas O'Dwyer, Catherine L Stables, Christopher Wood, Andrew D Brown, Victoria Speed, Lucy Bridges, Andrea L Schaffer, Caroline E Walters, Christopher T Rentsch, Krishnan Bhaskaran, Anna Schultze, Elizabeth J Williamson, Helen I McDonald, Laurie A Tomlinson, Rohini Mathur, Rosalind M Eggo, Kevin Wing, Angel Y S Wong, John Tazare, Richard Grieve, Daniel J Grint, Sinead Langan, Kathryn E Mansfield, Ian J Douglas, Stephen J W Evans, Liam Smeeth, Jemma L Walker, Viyaasan Mahalingasivam, Harriet Forbes, Thomas E Cowling, Emily L Herrett, Ruth E Costello, Bang Zheng, Edward P K Parker, Christopher Bates, Jonathan Cockburn, John Parry, Frank Hester, Sam Harper, Shaun O'Hanlon, Alex Eavis, Richard Jarvis, Dima Avramov, Paul Griffiths, Aaron Fowles, Nasreen Parkes, Brian Nicholson, Rafael Perera, David Harrison, Kamlesh Khunti, Jonathan A C Sterne, Jennifer Quint, Nishi Chaturvedi, Chloe Park, Alisia Carnemolla, Dylan Williams, Anika Knueppel, Andy Boyd, Emma L Turner, Katharine M Evans, Richard Thomas, Samantha Berman, Stela McLachlan, Matthew Crane, Rebecca Whitehorn, Jacqui Oakley, Diane Foster, Hannah Woodward, Kirsteen C Campbell, Nicholas Timpson, Alex Kwong, Ana Goncalves Soares, Gareth Griffith, Renin Toms, Louise Jones, Herbert Annie, Ruth Mitchell, Tom Palmer, Jonathan Sterne, Venexia Walker, Lizzie Huntley, Laura Fox, Rachel Denholm, Rochelle Knight, Kate Northstone, Arun Kanagaratnam, Elsie Horne, Harriet Forbes, Teri North, Kurt Taylor, Marwa A L Arab, Scott Walker, Jose I C Coronado, Arun S Karthikeyan, George Ploubidis, Bettina Moltrecht, Charlotte Booth, Sam Parsons, Bozena Wielgoszewska, Charis Bridger-Staatz, Claire Steves, Ellen Thompson, Paz Garcia, Nathan Cheetham, Ruth Bowyer, Maxim Freydin, Amy Roberts, Ben Goldacre, Alex Walker, Jess Morley, William Hulme, Linda Nab, Louis Fisher, Brian MacKenna, Colm Andrews, Helen Curtis, Lisa Hopcroft, Amelia Green, Praveetha Patalay, Jane Maddock, Kishan Patel, Jean Stafford, Wels Jacques, Kate Tilling, John Macleod, Eoin McElroy, Anoop Shah, Richard Silverwood, Spiros Denaxas, Robin Flaig, Daniel McCartney, Archie Campbell, Laurie Tomlinson, John Tazare, Bang Zheng, Liam Smeeth, Emily Herrett, Thomas Cowling, Kate Mansfield, Ruth E Costello, Kevin Wang, Kathryn Mansfield, Viyaasan Mahalingasivam, Ian Douglas, Sinead Langan, Sinead Brophy, Michael Parker, Jonathan Kennedy, Rosie McEachan, John Wright, Kathryn Willan, Ellena Badrick, Gillian Santorelli, Tiffany Yang, Bo Hou, Andrew Steptoe, Di Gessa Giorgio, Jingmin Zhu, Paola Zaninotto, Angela Wood, Genevieve Cezard, Samantha Ip, Tom Bolton, Alexia Sampri, Elena Rafeti, Fatima Almaghrabi, Aziz Sheikh, Syed A Shah, Vittal Katikireddi, Richard Shaw, Olivia Hamilton, Michael Green, Theocharis Kromydas, Daniel Kopasker, Felix Greaves, Robert Willans, Fiona Glen, Steve Sharp, Alun Hughes, Andrew Wong, Lee Hamill Howes, Alicja Rapala, Lidia Nigrelli, Fintan McArdle, Chelsea Beckford, Betty Raman, Richard Dobson, Amos Folarin, Callum Stewart, Yatharth Ranjan, Jd Carpentieri, Laura Sheard, Chao Fang, Sarah Baz, Andy Gibson, John Kellas, Stefan Neubauer, Stefan Piechnik, Elena Lukaschuk, Laura C Saunders, James M Wild, Stephen Smith, Peter Jezzard, Elizabeth Tunnicliffe, Zeena-Britt Sanders, Lucy Finnigan, Vanessa Ferreira, Mark Green, Rebecca Rhead, Milla Kibble, Yinghui Wei, Agnieszka Lemanska, Francisco Perez-Reche, Dominik Piehlmaier, Lucy Teece, Edward Parker, Rosemary J Hollick, Corri Black, Sinead Brophy, Ernest Choy, Gary Macfarlane, Louise Bennett, Lorna Philip, Michelle Stevenson, Denise McFarlane, Laura Moir, Ian Allotay, Philip Bell, Amanda Cheesley, Charlotte Marlow, Farzana Kausir, Emily Lam, Inga Wood

**Affiliations:** London School of Hygiene and Tropical Medicine, London, UK; National Centre for Population Health and Wellbeing Research, Swansea University Medical School, Wales, UK; National Centre for Population Health and Wellbeing Research, Swansea University Medical School, Wales, UK; National Centre for Population Health and Wellbeing Research, Swansea University Medical School, Wales, UK; Nuffield Department of Primary Care Health Sciences, Bennett Institute for Applied Data Science, University of Oxford, Oxford, UK; Nuffield Department of Primary Care Health Sciences, Bennett Institute for Applied Data Science, University of Oxford, Oxford, UK; Nuffield Department of Primary Care Health Sciences, Bennett Institute for Applied Data Science, University of Oxford, Oxford, UK; Nuffield Department of Primary Care Health Sciences, Bennett Institute for Applied Data Science, University of Oxford, Oxford, UK; Nuffield Department of Primary Care Health Sciences, Bennett Institute for Applied Data Science, University of Oxford, Oxford, UK; London School of Hygiene and Tropical Medicine, London, UK; Aberdeen Centre for Arthritis and Musculoskeletal Health (Epidemiology Group), University of Aberdeen, Health Sciences Building, Aberdeen, UK; Centre for Epidemiology Versus Arthritis, University of Manchester, Manchester Academic Health Centre, Manchester, UK

**Keywords:** rheumatoid arthritis, delivery of health care, inequalities, observational studies, organization of health care

## Abstract

**Objectives:**

We aimed to estimate how rheumatology outpatient hospital attendances have changed since the COVID-19 pandemic and determine demographic characteristics associated with observed changes.

**Methods:**

Using three primary and secondary care electronic health record datasets in England (with the approval of NHS England), Scotland and Wales, we identified people with a diagnosis of RA before 1 April 2019. We determined the proportion of people with rheumatology hospital outpatient appointments each month [April 2019 to December 2022 (Wales and Scotland), November 2023 (England)] and quantified changes using interrupted time-series analysis. We used logistic regression to determine characteristics associated with having fewer appointments compared with 2019.

**Results:**

We identified 145 065, 3813 and 13 637 people coded with RA in England, Scotland and Wales, respectively. At the start of the COVID-19 pandemic the number of rheumatology outpatient appointments dropped sharply across all nations. In England and Scotland, the percentage of monthly appointments has continued to decline. In Wales, while there was a gradual recovery, rheumatology services have not returned to pre-pandemic levels. In contrast, the number of appointments for other specialties has recovered in all nations. People with no rheumatology outpatient appointments were more often aged over 80, male and living in rural areas. Ethnic minorities, those living in more deprived and urban areas had fewer appointments after the start of the pandemic compared with 2019.

**Conclusion:**

For the first time, we compared healthcare use across three UK nations and found rheumatology outpatient appointments had not recovered to pre-COVID-19 pandemic levels, particularly in Scotland and England.

Rheumatology key messages

- Rheumatology outpatient appointments remain below pre-pandemic levels, particularly in Scotland and England, unlike other specialties.
- Ethnic minorities, deprived communities and urban residents had fewer rheumatology appointments post-pandemic than in 2019.
- Rheumatology services need data-driven strategies to provide better support, tailored to local community needs.

## Introduction

RA is a chronic inflammatory condition that requires ongoing specialist care. Rheumatologists employ a treat-to-target strategy aiming for minimal disease activity, achieved through frequent monitoring and adaptation of treatments [1]. In early inflammatory arthritis, monitoring every one to three months is recommended [2]. In the UK this can move to annual review once the disease is stable. This enables monitoring of disease activity and the development of comorbidities. Patients should have access to specialist care in the case of disease flare-ups [3].

Rheumatology services were significantly affected by the COVID-19 pandemic, with rheumatologists frequently seconded to help treat COVID-19 patients. Rheumatology and primary care appointments were moved to telephone or video consultations and drug monitoring was reduced [4, 5]. It is not clear how this impacted people with RA. There have been reports of medication interruptions during the pandemic, resulting in disease flares. However, many of these studies are based on self-reported data [6–8]. Beyond the impact of the pandemic, health inequities exist in RA, with greater deprivation being associated with worse outcomes [9, 10]. There is also evidence that people of lower socioeconomic status (SES), older age and those living in a rural location can struggle to access rheumatology services [11].

Prior to the COVID-19 pandemic, several countries have reported a shortage of rheumatologists, which is predicted to worsen [4, 12, 13]. The UK rheumatology workforce is understaffed. It is recommended that there is one consultant per 60 000 population; however, there is significant variation across the devolved nations. For example, there is one consultant per 80 617 in England and one per 111 637 in Scotland, resulting in potential gaps in care [4].

Furthermore, in the UK, healthcare is delivered under the umbrella of the National Health Service (NHS); however, health is devolved across the four nations of the UK (England, Wales, Scotland and Northern Ireland). Each nation has variation within healthcare systems, with divergent policies and priorities.

We had access to linked primary and secondary electronic health record (EHR) data from three of the four nations (England, Wales and Scotland) providing a unique opportunity to explore healthcare use, specifically rheumatology outpatient attendances, across devolved nations of the UK and examine the differential impacts since the COVID-19 pandemic. In patients with RA we aimed to (i) estimate how hospital outpatient appointments have changed over the course of the pandemic, compared with 2019; (ii) determine socio-demographic characteristics associated with any observed changes; and (iii) compare changes in rheumatology outpatient attendances with changes in other specialties.

## Methods

### Study design and data sources

We conducted a population-based observational cohort study of people with RA using electronic health records (EHR) in England, Wales and Scotland.

In England, we used primary care records managed by the GP software provider TPP linked to outpatient appointment data from the Hospital Episode Statistics for England and mortality data from the Office for National Statistics (ONS) through OpenSAFELY. TPP patients represent 42.5% of the England population and are broadly representative [14]. All data were linked, stored and analysed securely within the OpenSAFELY platform: https://opensafely.org/, as part of the NHS England OpenSAFELY COVID-19 service. This includes pseudonymized data such as coded diagnoses, medications and physiological parameters. Free text data are excluded. All code is shared openly for review and re-use under MIT open licence (https://github.com/opensafely/RA_outcomes). Detailed pseudonymized patient data is potentially re-identifiable and therefore not shared.

Primary care data for around 85% of the Welsh population is available within the SAIL Databank. All people alive in Wales registered with a general practice who contribute data to SAIL were identified as of 23 March 2020. Individuals with diagnostic codes for RA from 1 January 2005 to 22 March 2020 were identified from this general population group using primary care records in the Welsh Longitudinal General Practice (WLGP) database. This information was linked to other national databases in SAIL including: outpatient appointments, emergency care and hospital admissions [15].

In contrast, there is no national, anonymized primary care dataset in Scotland and primary care data can only be accessed through a trusted third-party provider. This process currently requires written permission from individual GP practices and reimbursement for time to complete the agreements. Due to time and financial constraints, this limited the scope of data that could be included from Scotland. We report on primary care data from two health boards in Scotland: Grampian and Highland. These were selected to provide a mix of urban, accessible and remote rural mainland communities and island communities and different healthcare settings. A total of 50% of practices approached granted permission and their data were included in the study. Primary care data was linked to other national databases in Public Health Scotland, including outpatient hospital appointments, hospital admission records, registries for death and cancer, and all medications dispensed in community care. Data linkage was conducted by the NHS Scotland electronic Data Research and Innovation Service via deterministic linkage methods using unique personal identification numbers in a process shown to produce highly accurate and complete data [16]. Data was accessed through the National Data Safehaven.

In England, this study was approved by the Health Research Authority (REC reference 20/LO/0651) and by the LSHTM Ethics Board (reference 21863). In Wales, the study was approved by the SAIL Information Governance Review Panel (approval number: 0419). All data used in this study can be accessed by request to SAIL. Approvals for data linkage in Scotland were obtained from the Public Benefit and Privacy Panel for Health and Social Care, Scotland (reference number 1819–0286). More information on ethics can be found in the disclosures section.

### Study population

Our study start date was 1 April 2019, based on data availability across all three datasets. We identified people with coded RA on or prior to 1 April 2019 (index date) using a validated algorithm [17]. The algorithm uses diagnosis (Read or SNOMED CT codes) and prescription codes from the primary care EHRs. In England and Wales people were considered to have a diagnosis of RA if they had either (i) ≥2 RA diagnosis codes on different dates with at least one ‘strong’ codes indicating seropositive or erosive RA or specifically RA as opposed to systemic manifestations, seronegative RA or other weak evidence of RA, and no alternative diagnosis; or (ii) a single RA diagnosis code and disease-modifying anti-rheumatic drug (DMARD) prescribing after the first RA code and no alternative indication. In Scotland, due to insufficient prescribing data, this was based on the presence of ≥2 RA codes. We required people to have at least 3 months registration with their GP prior to 1 April 2019 and be aged 18–115 years. We excluded people with missing age, sex, region or index of multiple deprivation as this could indicate poor data quality. We followed people until the earliest of death, deregistration from their practice or the end of the study period (Wales and Scotland: 31 December 2022, England: 30 November 2023).

### Study measures

Using these cohorts of people with a diagnosis of RA, we identified all hospital outpatient appointments occurring during the study period in the secondary care data. We then classified these by whether they were with the rheumatology specialty or other medical and surgical specialties. Thus, reported rates of outpatient appointments for rheumatology and non-rheumatology specialties refer specifically to individuals with RA. In England these were appointments attended; in Scotland and Wales, these were appointments scheduled.

We determined the following socio-demographic characteristics at index date: age, sex, ethnicity, urban-rural classification and country-specific index of multiple deprivation (IMD), an area-based measures of deprivation (Table 1). We identified smoking status at index date by identifying the latest smoking code and any record of prior smoking, and classified people as either non-smokers, ex-smokers or current smokers. BMI at baseline was defined using the most recent measurement within the 5 years prior to baseline.

**Table 1.** Covariate definitions for each nation

	England	Wales	Scotland
Region	9 regions of England based on NHS administrative geographical areas	22 Local Authorities	2 Health Boards (Highlands and Grampian)
Rural–urban classification	Based on Rural Urban Classification for England and Wales [18] and categorised into urban and rural	Based on Rural Urban Classification for England and Wales [18] and categorised into urban and rural	Based on Rural Urban Classification for Scotland [19] and categorised into urban and rural
Deprivation	Quintiles of Index of Multiple Deprivation (IMD) based on a person’s postcode [20]	Quintiles based on the Welsh Index of Multiple Deprivation (WIMD) based on 1990 LSOAs [21]	Quintiles based on the Scottish Index of Deprivation (SIMD) based on 6976 data zones [22]
Ethnicity	Self-reported ethnicity identified using SNOMED CT codes in primary care record, supplemented with ethnicity recorded in the secondary care record	Self-reported ethnicity identified using SNOMED CT codes in primary care record, supplemented with ethnicity recorded in the secondary care record	Not available

### Statistical analysis

We describe the baseline socio-demographic characteristics and healthcare use of each nation. For outpatient appointments with rheumatology and all other hospital-based specialties, we determined the proportion of people who attended/had scheduled appointments each month. We quantify changes using time-series analysis, where monthly proportions were modelled in an ordinary least-squares regression model with Newey-West heteroskedasticity-consistent standard errors and one lag to account for autocorrelation, determined through model checks including autoregression and partial autocorrelation plots. The interruption was set at 23 March 2020. To further understand how appointments changed over time, and because frequently people are seen on an annual basis in rheumatology, we counted the number of outpatient appointments with (i) rheumatology and (ii) all other specialties each year: April–March. People were classified as having either zero, 1–2 or ≥3 rheumatology outpatient appointments in a given year. We excluded people from these counts if they were deregistered with a general practice, or died during the year of interest as their count would not represent the whole year. We compared counts in each of the years during and after the COVID-19 pandemic (starting April 2020 onwards) to counts in the year prior to the pandemic (year starting April 2019). We then determined if people had either no appointments in a year, fewer appointments compared with the pre-pandemic year or the same number or more appointments compared with the pre-pandemic year. In people who had appointments in both comparator years, we described the socio-demographic characteristics of these groups and used univariate and multivariate logistic regression models to measure whether age, sex, ethnicity, rural-urban classification area-based measure of deprivation (IMD) were associated with having fewer appointments compared with 2019. The aim was to identify whether specific demographic groups were less likely to have maintained pre-pandemic levels of care, which could indicate inequitable access to services during or following the pandemic.

The pre-specified variables included in the logistic regression models were determined *a priori* through discussion with the clinical and analytical teams.

### Missing data

A missing category was used to indicate where covariates were missing, including BMI, smoking and ethnicity. As there was a large amount of missing data for ethnicity in Wales, for descriptive purposes ethnicity was categorized as White, Ethnic Minorities or Unknown, and ethnicity was not included in the logistic regression models.

### Software and reproducibility

Data management in England was performed using Python 3.9.7, with analysis carried out using Stata 17. Code for data management and analysis, as well as codelists and the protocol, are archived online (https://github.com/opensafely/RA_outcomes/tree/main).

Data management in Wales was primarily conducted on an IBM database (DB2) using SQL. Scottish data was received as comma-separated-value files, which were imported into RStudio 4.4.1 for handling. In Wales and Scotland analysis was performed using RStudio 4.4.1. Data visualizations combining all nation data were created in RStudio using ggplot2 and other open-source libraries.

## Results

### Demographics

The number of people with RA on 1 April 2019 in the three nation cohorts was as follows: 145 065 (England), 3813 (Scotland) and 13 637 (Wales). The English Cohort was older [age >80 years: 15.9% (England), 11.7% (Wales) and 12.7% (Scotland)], with fewer people living in rural areas than Scotland or Wales [rural: 24.7% (England), 32.0% (Wales) and 33.7% (Scotland)]. The Scottish cohort was less deprived than England and Wales [IMD 1 (most deprived): 17.9% (England), 19.3% (Wales), 6.4% (Scotland)]. The English cohort had a longer disease duration than Wales and Scotland [time since first RA code: 12.2 years (England), 9.5 years (Wales), 7.6 years (Scotland)] (Table 2).

**Table 2.** Characteristics of each cohort at study start (1 April 2019)

		England^a^	Wales	Scotland
Number of people		*N* = 145,065	*N* = 13,637	*N* = 3,813
				
Age, *n* (%)	18-40 years	7910 (5.5)	775 (5.7)	279 (7.3)
	41-60 years	39305 (27.1)	4055 (29.7)	1174 (30.8)
	61-80 years	74805 (51.6)	7258 (53.2)	1877 (49.2)
	80+ years	23045 (15.9)	1549 (11.4)	483 (12.7)
				
Sex, *n* (%)	Female	102145 (70.4)	9533 (69.9)	2557 (67.1)
	Male	42925 (29.6)	4104 (30.1)	1256 (32.9)
				
Rural-Urban classification, *n* (%)	Rural	35825 (24.7)	4362 (32.0)	2527 (66.3)
	Urban	109245 (75.3)	9275 (68.0)	1286 (33.7)
				
Index of Multiple Deprivation, *n* (%)	1 (most deprived)	25995 (17.9)	2637 (19.3)	245 (6.4)
	2	28310 (19.5)	3037 (22.3)	601 (15.8)
	3	32695 (22.5)	2871 (21.1)	1046 (27.4)
	4	30375 (20.9)	2605 (19.1)	1338 (35.1)
	5 (Least deprived)	27695 (19.1)	2487 (18.2)	583 (15.3)
				
Smoking, *n* (%)	Never	53555 (36.9)	3496 (25.6)	–
	Current	20390 (14.1)	3108 (22.8)	–
	Former	70740 (48.8)	7033 (51.6)	–
	Missing	380 (0.3)	–	–
				
BMI, *n* (%)	Underweight	3605 (2.5)	–	–
	Healthy weight	38440 (26.5)	–	–
	Overweight	42120 (29)	–	–
	Obese	34375 (23.7)	–	–
	Severe obesity	6250 (4.3)	–	–
	Missing	20280 (14)	–	–
				
Ethnicity, *n* (%)	White	131975 (91)	6227 (45.7)	–
	Asian	8210 (5.7)	–	–
	Black	1910 (1.3)	–	–
	Mixed	890 (0.6)	–	–
	Other	1085 (0.7)	–	–
	Ethnic minorities		78 (0.6)	–
	Missing	1000 (0.7)	7332 (53.8)	–
				
Time since first RA code (years), mean (SD)		12.21 (10.97)	9.49 (5.63)	7.63 (5.01)

a Counts for England rounded to the nearest 5.

### Outpatient hospital appointments per month

Rheumatology appointments

In England and Wales on average 14% of the RA cohorts attended a rheumatology outpatient appointment each month in the year prior to the COVID-19 pandemic. In Scotland this figure was 8%. Across all nations there was a marked reduction in the proportion of the cohort having rheumatology appointments at the start of the pandemic (March 2020).

**Figure 1.**
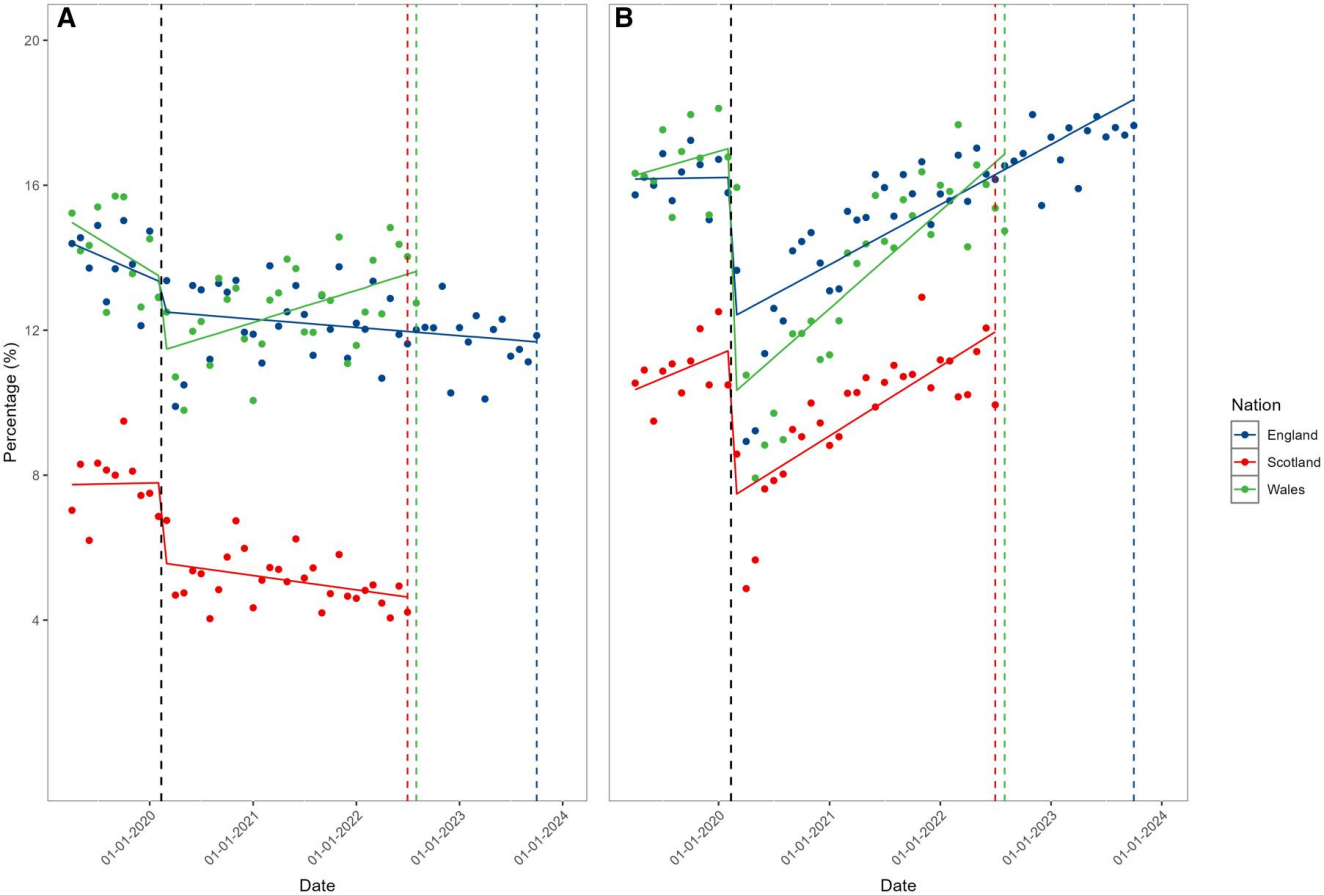
Percentage of the cohorts to attending (England)/scheduled to attend (Scotland/Wales) rheumatology (**A**) or all other (**B**) outpatient appointments each month. Black dotted line indicates the beginning of lockdown restrictions in the UK. Coloured dotted lines indicate the end of the study period in each nation

In Wales, the proportion of the cohort with a rheumatology outpatients appointment then increased from 12% in June 2020 to13% in August 2022. In Scotland and England during the same period, the proportion with a rheumatology appointment each month continued to decrease over time from 5.4% to 4.2% in Scotland, and from 13% to 11% in England (Fig. 1).

Non-rheumatology appointments

For other speciality outpatient appointments attended by the RA cohorts, a similar reduction in appointments was seen at the start of the pandemic; however, appointment frequency recovered to at least pre-pandemic levels in all nations by the end of the study period (Fig. 1).

### Trends in outpatient hospital appointments per year

Rheumatology appointments

England and Wales had similar patterns in the proportion of people with RA attending or scheduled to attend rheumatology appointments per year. In the year prior to the COVID-19 pandemic (April 2019 to March 2020), people most frequently had one to two rheumatology outpatient appointments per year (England: 44%, Wales: 46%), with around one-third having no appointments (England: 30%, Wales: 27%) and a quarter having three or more appointments per year (England: 25%, Wales 27%).

This pattern continued after the start of the pandemic; however, there was a higher proportion of people who had no appointments [year starting April 2020: 33.6% (England), 32.5% (Wales)]. When comparing 2020–2019, 22% of the cohort in England and 18% of the cohort in Wales had no rheumatology appointments in either year; 35% of the cohort in England and 38% of the cohort in Wales had fewer appointments.

In Scotland, in the year prior to the COVID-19 pandemic, 47% of the cohort had no rheumatology outpatient appointments, 44% had one to two appointments and 9% had three or more appointments. When comparing 2020–2019, the proportion of the cohort with no appointments increased to 63%; 41% had no appointments in either year and 33% had fewer appointments (Table 3).

**Table 3.** Number of rheumatology outpatient appointments in comparison to 2019, across each nation and each year

		April 2019–March 2020			April 2020–March 2021			April 2021–March 2022			April 2022–March 2023		
		England	Wales	Scotland	England	Wales	Scotland	England	Wales	Scotland	England	Wales	Scotland
Number of people^a^		138 835	13 021	3614	132 085	12 351	3412	125 640	11 791	3238	119 295	–	–
Number of rheumatology appointments, *n* (%)	Zero appointments	42 180 (30.4)	3 498 (26.9)	1705 (47.2)	44 430 (33.6)	4 014 (32.5)	2146 (62.9)	42 450 (33.8)	4 204 (35.7)	2135 (65.9)	41 245 (34.6)	–	–
	1-2 per year	61 705 (44.4)	5 947 (45.7)	1567 (43.4)	59 160 (44.8)	5 350 (43.3)	1039 (30.5)	55 360 (44.1)	4 441 (37.7)	852 (26.3)	53 210 (44.6)	–	–
	3 or more per year	34 950 (25.2)	3 576 (27.5)	342 (9.5)	28 495 (21.6)	2 987 (24.2)	227 (6.7)	27 830 (22.2)	3 146 (26.7)	251 (7.8)	24 840 (20.8)	–	–
													
Difference in number of rheumatology appointments compared to 2019, *n* (%)	Zero appointments both years	–	–	–	28 905 (21.9)	2 271 (18.4)	1411 (41.4)	26 490 (21.1)	2 127 (17.2)	1356 (41.9)	24 245 (20.3)	–	–
	Fewer appointments	–	–	–	45 555 (34.5)	4 666 (37.8)	1141 (33.4)	44 675 (35.6)	4 461 (36.1)	1106 (34.2)	45 235 (37.9)	–	–
	Same number or more appointments	–	–	–	57 625 (43.6)	5 414 (43.8)	860 (25.2)	54 480 (43.4)	5 203 (42.1)	776 (24.0)	25 570 (41.8)	–	–

a Counts for England rounded to the nearest 5.

Non-rheumatology specialist appointments

In England and Wales, the majority of people with RA had six or more non-rheumatology outpatient appointments per year in the year before the COVID-19 pandemic; in Scotland, most of the cohort had between one and two appointments. A small proportion of the cohort did not have any non-rheumatology specialist outpatient appointments: 14.8% in England, 12.4% in Wales and 25.8% in Scotland.

Compared with 2019, in 2020, the majority of people with RA in all three countries had one to two appointments: 31.3% in England, 33.6% in Wales and 34.2% in Scotland. By 2021, patterns in England and Wales had shifted back towards pre-pandemic levels, with most patients having six or more appointments. In contrast, in Scotland the largest proportion of patients (30.1%) still had only one to two appointments (Supplementary Table S1).

### Differences in healthcare use by sociodemographic characteristics

Across all nations, those with no scheduled or attended rheumatology outpatient hospital appointments were more likely to be older than 80 years, male and live in a rural area (Supplementary Tables S2 and S3).

**Figure 2.**
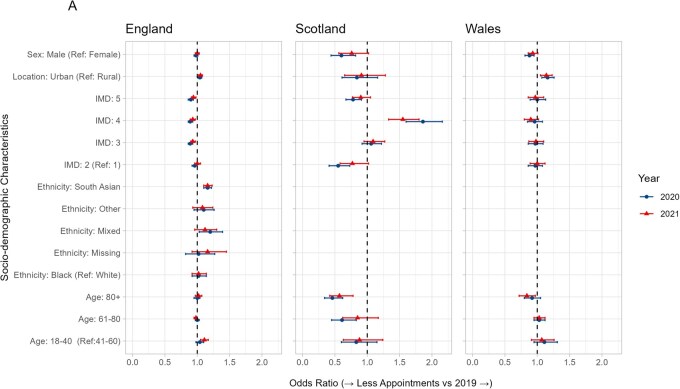
Forest plot of univariate odds ratios and 95% confidence intervals for having fewer rheumatology outpatient appointments in 2020 and 2021 compared with 2019, by sociodemographic characteristics. An odds ratio above one indicates that people were more likely to have fewer appointments, and odds ratio below one indicates the people were less likely to have fewer appointments

**Figure 3.**
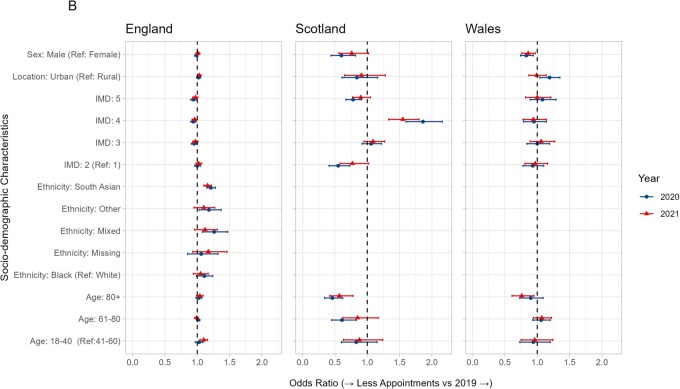
Forest plot of multivariate odds ratios and 95% confidence intervals for having fewer rheumatology outpatient appointments in 2020 and 2021 compared with 2019, by sociodemographic characteristics. An odds ratio above one indicates that people were more likely to have fewer appointments, and odds ratio below one indicates that people were less likely to have fewer appointments

For those with outpatient hospital appointments, in England, the most deprived were more likely to have fewer appointments in 2020 than 2019 [IMD 5 (least deprived): OR 0.94, 95% CI 0.90–0.98, IMD 4: OR 0.94, 95% CI 0.90–0.98. IMD 3: OR 0.95, 95% CI 0.91–0.99] (Figs 2 and 3, Supplementary Table S4). A similar pattern was seen in Wales and Scotland, though with fewer individuals, there was greater uncertainty around the estimates.

In Wales and Scotland, people living in an urban area were more likely to have fewer appointments [OR (95% CI): 1.19 (1.04, 1.35) (Wales), 1.70 (1.44, 2.00) (Scotland)]; there was little difference in England [OR 95% CI: 1.02 (0.99, 1.05)]. In Wales and Scotland, males were less likely to have fewer appointments [OR 95% CI: 0.83 (0.74, 0.94) (Wales), 0.81 (0.69, 0.95) (Scotland)], but this was not seen in England [OR 95% CI: 0.99 (0.96, 1.01)]. In England, those with Mixed, Asian or Other ethnicity were more likely to have had fewer outpatient appointments (Asian: OR 1.21, 95% CI 1.17, 1.28, Mixed: OR 1.26, 95% CI 1.08, 1.47, Other: OR 1.18, 95% CI 1.03, 1.37). Similar patterns were seen when comparing 2021–2019 (Supplementary Table S5).

## Discussion

This is the first study to compare outpatient hospital appointments and socio-demographic characteristics of people with RA in the three devolved nations of the UK during the COVID-19 pandemic and the recovery period. We found a reduction in the proportion of rheumatology outpatient appointments at the start of the pandemic in all three nations; however, subsequent recovery varied. Since June 2020, in Wales, there has been a recovery in scheduled rheumatology appointments, albeit not to pre-pandemic levels. By contrast, in England and Scotland rheumatology outpatient appointments offered or attended had not recovered at the end of the study period. The proportion of patients with rheumatology appointments reduced over time into 2022 in Scotland and into 2023 in England. A high proportion of patients were not seen for several years, particularly in Scotland where 41% were not seen in 2019 and 2020. These people were frequently over 80 years old. In England, ethnic minorities and those living in areas with higher deprivation were more likely to have fewer appointments after the start of the pandemic.

This study provides a unique perspective across three UK nations on healthcare utilization for an exemplar long-term condition requiring regular hospital follow-up. It highlights the context of devolved healthcare systems with differing priorities and policies before and after the COVID-19 pandemic.

There are some limitations to the study. We did not have complete population coverage, potentially affecting representativeness. For example Scottish data were limited to two health boards which are more rural and less deprived than the Scottish average. However, our primary care cohorts had an estimated 0.5% to 0.8% prevalence of RA; this is in keeping with national estimates, typically 0.5% to 1% of adults in developed countries [23]. In Wales and Scotland, hospital outpatient records described appointments scheduled, whereas in England attended appointments rates were available. This may explain the higher number of appointments in Wales; however, the non-attendance rates are low in rheumatology [24] and likely to be distributed evenly across the cohorts. The algorithm defining RA cases included primary care prescribing of csDMARDs. As this represents shared care prescribing, it may be an underestimation in Wales and England. However, whilst initiation of csDMARDs may take place in secondary care in many settings, ongoing prescriptions are provided by primary care. Therefore any underestimate is likely to be small. We used a linear time-series model to measure change in appointments after the start of the pandemic, which is limited by only fitting linear trend lines, as opposed to a more flexible model. However, we had no reason to believe that the trends would not be linear. There were no measures of disease severity in this data which meant we could not determine whether people with fewer appointments had better disease control. Some information was unavailable, poorly documented or complex to include, such as smoking and ethnicity, which meant we could not fully investigate their impact across all countries. Certain variables, including rural-urban classification, IMD and ethnicity, may also be interrelated, though this was outside the scope of this study.

Studies of the impact of the pandemic service provision in people with RA have generally focused on 2020, and most have been survey based. Studies, in the UK, Europe, the US and Egypt have indicated that patients felt they had reduced access to care during the pandemic [11, 25–29]. In a survey of patients in the UK, only 4.5% had not seen a rheumatologist at all over 18 months, which is lower than our study, but may reflect selection bias in who completed the survey [26].

A Royal College of Physicians survey in July 2020, found that physicians across a range of specialities did not expect to be back at full operational capacity within 12 months, even without any further major pandemic events. Specifically, rheumatologists expected to be back to 75% capacity [30]. Our results suggest that the recovery of rheumatology services has been slow. Potential reasons for the slow recovery of rheumatology services post COVID-19 pandemic are multi-factorial. Firstly, rheumatology services were under considerable strain prior to the COVID-19 pandemic [4]. The COVID-19 pandemic put further strain on already stressed rheumatology services, where many rheumatologists were deployed to frontline COVID-19 care in the early months. As a result, waiting lists lengthened. The finding of such a high proportion of RA patients with no rheumatology appointments is striking and clinically important. There are two potential explanations for this: (i) data-related factors such as misclassification of diagnosis and (ii) clinical or service-related factors. During the COVID-19 pandemic there was substantial disruption to planned care, even before the first official UK lockdown. Non-urgent appointments, particularly for patients considered stable, were frequently postponed. Our data suggest that rheumatology services did not return to pre-pandemic activity levels, unlike many other specialties, indicating that these service pressures and deferrals persisted well beyond the initial pandemic response.

Workforce data provides important contextual insights. According to the British Society for Rheumatology (BSR) 2021 workforce report [4], all nations have below the recommended minimum consultant rheumatologist : population ratio. Scotland has the lowest, with just one consultant per 111 637 people. In addition to workforce shortages, the geography of the Scottish health boards included in our study – many of which cover remote, rural and island communities – poses further challenges for service delivery. These factors may help explain the lower rate of appointments observed in Scotland and highlight the importance of considering both workforce capacity and geographical context when interpreting variations in access to care.

Interestingly, this may be a rheumatology-specific finding, as our data showed that the amount of outpatient hospital appointments in other specialities attended by our RA cohorts have recovered to at least pre-pandemic levels. It is not unexpected that a higher proportion of individuals had at least one non-rheumatology outpatient appointment, given that RA is associated with several comorbidities. However, the most striking finding that these returned to pre-pandemic level, unlike the number of rheumatology outpatient appointments, which continued to decline.

This was mirrored NHS England’s Hospital Outpatient Activity statistical publications 2018–19 to 2023–24 [31], which is not limited to patients with RA. While outpatient activity in most medical specialties (e.g. nephrology, respiratory medicine, gastroenterology, medical oncology) has remained stable or increased post-pandemic, rheumatology outpatient appointments (agnostic of diagnosis) have consistently declined since 2020/21. While the percentage differences may appear small, they translate into a substantial absolute number of appointments. For example, in 2023/24 there were a total of 1 768 016 rheumatology outpatient appointments in England [31]. Therefore, a 2% reduction in outpatient appointments for people with RA, reflected across the wider rheumatology population, equates to ∼35 360 fewer appointments per year. This represents a significant shortfall in service provision.

The association between ethnic minorities and higher deprivation (IMD) with fewer outpatient appointments post-pandemic is particularly concerning, given the fact that ethnic minority groups and individuals living in more deprived areas not only experienced worse outcomes during the COVID-19 pandemic but also tend to have poorer health outcomes in rheumatic conditions more generally [32].

## Implications

Rheumatic and musculoskeletal disease care has historically not been a policy priority, and this is reflected in the reduction in rheumatology outpatient appointments for people with RA, in contrast to the recovery of appointments observed in other specialties following the COVID-19 pandemic. People with RA would typically expect to see a member of the rheumatology multidisciplinary team at least once a year as part of ongoing disease management. The overall decline in appointments observed, along with the substantial proportion of individuals who were not seen at all for a year or more, likely represents missed opportunities for timely assessment and intervention, potentially leading to worse long-term outcomes [33].

Our findings provide the opportunity for cross-border learning in terms of data-driven approaches to service planning and access to primary and secondary care health data to support this, as well as evaluation of differing policy approaches across the devolved nations. We have identified groups of individuals that are less likely to be seen in rheumatology clinics since the start of the pandemic. The wider RHEUMAPS study has used the data to create interactive geo-spatial maps in Wales and Scotland to support local, regional and national service planning [34].

## Conclusions

Access to specialist rheumatology care for people with RA reduced at the start of the COVID-19 pandemic and had not recovered by the end of the study periods in England and Scotland; whilst Wales has shown some recovery. There are groups of people with RA who are missing out on specialist care, representing missed opportunities for management of RA and potential for poorer long-term outcomes. These findings highlight the importance of tailored responses to different populations of the UK, particularly in times of – or response to – significant healthcare stressors, to ensure equitable care.

## Ethics and information governance

In England, this study was approved by the Health Research Authority (REC reference 20/LO/0651) and by the LSHTM Ethics Board (reference 21863).

In Wales, the study was approved by the SAIL Information Governance Review Panel (approval number: 0419). All data used in this study can be accessed by request to SAIL.

Approvals for data linkage in Scotland were obtained from the Public Benefit and Privacy Panel for Health and Social Care, Scotland (reference number 1819–0286).

In England, NHS England is the data controller of the NHS England OpenSAFELY COVID-19 Service; TPP is the data processor; all study authors using OpenSAFELY have the approval of NHS England [35]. This implementation of OpenSAFELY is hosted within the TPP environment which is accredited to the ISO 27001 information security standard and is NHS IG Toolkit compliant [36].

Patient data has been pseudonymized for analysis and linkage using industry-standard cryptographic hashing techniques; all pseudonymized datasets transmitted for linkage onto OpenSAFELY are encrypted; access to the NHS England OpenSAFELY COVID-19 service is via a virtual private network (VPN) connection; the researchers hold contracts with NHS England and only access the platform to initiate database queries and statistical models; all database activity is logged; only aggregate statistical outputs leave the platform environment following best practice for anonymization of results such as statistical disclosure control for low cell counts [1].

The service adheres to the obligations of the UK General Data Protection Regulation (UK GDPR) and the Data Protection Act 2018. The service previously operated under notices initially issued in February 2020 by the Secretary of State under Regulation 3(4) of the Health Service (Control of Patient Information) Regulations 2002 (COPI Regulations), which required organizations to process confidential patient information for COVID-19 purposes; this set aside the requirement for patient consent [2]. As of 1 July 2023, the Secretary of State has requested that NHS England continue to operate the Service under the COVID-19 Directions 2020 [3]. In some cases of data sharing, the common law duty of confidence is met using, for example, patient consent or support from the Health Research Authority Confidentiality Advisory group [4].

Taken together, these provide the legal bases to link patient datasets using the service. GP practices, which provide access to the primary care data, are required to share relevant health information to support the public health response to the pandemic, and have been informed of how the service operates.

1. NHS Digital [Internet]. [cited 2023 Sep 20]. ISB1523: Anonymisation Standard for Publishing Health and Social Care Data. Available from: https://digital.nhs.uk/data-and-information/information-standards/information-standards-and-data-collections-including-extractions/publications-and-notifications/standards-and-collections/isb1523-anonymisation-standard-for-publishing-health-and-social-care-data
2. GOV.UK [Internet]. 2022 [cited 2023 Sep 20]. [Withdrawn] [withdrawn] Coronavirus (COVID-19): notice under regulation 3(4) of the Health Service (Control of Patient Information) Regulations 2002—general. Available from: https://www.gov.uk/government/publications/coronavirus-covid-19-notification-of-data-controllers-to-share-information/coronavirus-covid-19-notice-under-regulation-34-of-the-health-service-control-of-patient-information-regulations-2002-general—2
3. NHS Digital [Internet]. [cited 2023 Sep 20]. COVID-19 Public Health Directions 2020. Available from: https://digital.nhs.uk/about-nhs-digital/corporate-information-and-documents/directions-and-data-provision-notices/secretary-of-state-directions/covid-19-public-health-directions-2020
4. Health Research Authority [Internet]. [cited 2023 Sep 20]. Confidentiality Advisory Group. Available from: https://www.hra.nhs.uk/about-us/committees-and-services/confidentiality-advisory-group/

## Supplementary Material

keaf559_Supplementary_Data

## Data Availability

In England, access to the underlying identifiable and potentially re-identifiable pseudonymized electronic health record data is tightly governed by various legislative and regulatory frameworks and restricted by best practice. The data in the NHS England OpenSAFELY COVID-19 service is drawn from General Practice data across England where TPP is the data processor. TPP developers initiate an automated process to create pseudonymized records in the core OpenSAFELY database, which are copies of key structured data tables in the identifiable records. These pseudonymized records are linked onto key external data resources that have also been pseudonymized via SHA-512 one-way hashing of NHS numbers using a shared salt. University of Oxford, Bennett Institute for Applied Data Science developers and PIs, who hold contracts with NHS England, have access to the OpenSAFELY pseudonymized data tables to develop the OpenSAFELY tools. These tools in turn enable researchers with OpenSAFELY data access agreements to write and execute code for data management and data analysis without direct access to the underlying raw pseudonymized patient data, and to review the outputs of this code. All code for the full data management pipeline – from raw data to completed results for this analysis – and for the OpenSAFELY platform as a whole is available for review at github.com/OpenSAFELY. Access to NHS Scotland health data is governed by the NHS Scotland Public Benefit and Privacy Panel for Health and Social Care (HSC-PBPP). Access to the underlying pseudonymized health data used in this study is by application to the HSC-PBPP panel. All data used in this study can be accessed by request to SAIL. The data management and analysis code for this paper was led by Ruth E Costello in England and Michael Parker in Wales and Scotland.
