# Supplementary material for: Outpatient hospital attendances in people with rheumatoid arthritis during the COVID-19 pandemic and beyond: a cohort study in three nations of the UK
Source: Rheumatology (Oxford). 2025 Oct 24;65(1):keaf559. doi: 10.1093/rheumatology/keaf559 (PMC12862379; doi:10.1093/rheumatology/keaf559)
Supplement: keaf559_Supplementary_Data [file keaf559_supplementary_data.docx]

Supplementary materials

Contents

[Supplementary Table S1 Number of other specialty outpatient appointments, and comparison to 2019, by nation and year. 2](#_Toc205469874)

[Supplementary Table S2: Characteristics of patients by category of difference in the number of rheumatology outpatient appointments in 2020 compared with 2019 3](#_Toc205469875)

[Supplementary Table S3: Characteristics of patients by category of difference in the number of rheumatology outpatient appointments in 2021 compared with 2019 5](#_Toc205469876)

[Supplementary Table S4: Logistic regression results measuring characteristics of those who had fewer appointments in 2020 compared to 2019. 8](#_Toc205469877)

[Supplementary Table S5: Logistic regression measuring characteristics of those who had fewer appointments in 2021 compared to 2019. 9](#_Toc205469878)

[Supplementary Table S6: OpenSAFELY Collaborative 11](#_Toc205469879)

[Supplementary Table S7: The LH&W NCS Collaborative 12](#_Toc205469880)

[Supplementary text S8: RHEUMAPS study investigators 13](#_Toc205469881)

[Supplementary table S9: RECORD checklist 14](#_Toc205469882)

##

## Supplementary Table S1 Number of other specialty outpatient appointments, and comparison to 2019, by nation and year.

|  | | April 2019-March 2020 | | | April 2020 – March 2021 | | | April 2021 – March 2022 | | | April 2022 - March 2023 | | |
| --- | --- | --- | --- | --- | --- | --- | --- | --- | --- | --- | --- | --- | --- |
|  |  | England | Wales | Scotland | England | Wales | Scotland | England | Wales | Scotland | England | Wales | Scotland |
| Number of individuals* | | 138835 | 13021 | 3614 | 131195 | 12351 | 3412 | 124730 | 11791 | 3238 | 119295 | - | - |
| Number of All Appointments, n (column %) | Zero appointments | 20,570 (14.8) | 1,609 (12.36) | 933 (25.8) | 22,490 (17) | 2,278 (18.44) | 1384 (40.6) | 19,065 (15.2) | 2,042 (17.32) | 1256 (38.8) | 17,485 (14.7) | - | - |
|  | 1-2 per year | 34,830 (25.1) | 3,584 (27.52) | 1401 (38.8) | 41,380 (31.3) | 4,144 (33.55) | 1166 (34.2) | 35,190 (28) | 3,156 (26.77) | 975 (30.1) | 32,645 (27.4) | - | - |
|  | 3-5 per year | 35,655 (25.7) | 3,839 (29.48) | 844 (23.4) | 33,455 (25.3) | 3,452 (27.95) | 574 (16.8) | 31,410 (25) | 3,171 (26.89) | 615 (19.0) | 30,485 (25.6) | - | - |
|  | 6 or more per year | 47,780 (34.4) | 3,989 (30.64) | 436 (12.1) | 34,750 (26.3) | 2,477 (20.06) | 288 (8.4) | 39,980 (31.8) | 3422 (29.02) | 392 (12.1) | 38,680 (32.4) | - | - |
|  |  |  |  |  |  |  |  |  |  |  |  |  |  |
| Difference in number of Appointments compared to 2019, n (column %) | Zero appointments both years | - | - | - | 10,415 (7.9) | 899 (7.3) | 641 (18.8) | 8,425 (6.17) | 725 (5.87) | 561 (17.3) | 7,280 (6.1) | - | - |
|  | Fewer appointments | - | - | - | 65,930 (49.9) | 6,469 (54.8) | 1625 (47.6) | 56,480 (45.0) | 5,262 (42.6) | 1411 (43.6) | 52,895 (44.3) | - | - |
|  | Same number or more appointments | - | - | - | 55,735 (42.2) | 4,983 (40.3) | 1146 (33.6) | 60,740 (48.3) | 5,804 (46.99) | 1266 (39.1) | 59,120 (49.6) | - | - |

* Counts for England rounded to the nearest 5.

## Supplementary Table S2: Characteristics of patients by category of difference in the number of rheumatology outpatient appointments in 2020 compared with 2019

|  | | No appointments either year | | | Fewer appointments | | | Same number or more appointments | | |
| --- | --- | --- | --- | --- | --- | --- | --- | --- | --- | --- |
| Country | | England | Wales | Scotland | England | Wales | Scotland | England | Wales | Scotland |
| Number of individuals | | 28,895 | 2271 | 1403 | 45,530 | 4666 | 1139 | 57,580 | 5414 | 860 |
| Age category, n (row %) | 18 - 40 years | 1360 (18) | 116 (15.5) | 123 (48.4) | 2790 (36.9) | 298 (39.9) | 63 (24.8) | 3405 (45.1) | 332 (44.5) | 68 (26.8) |
|  | 41 - 60 years | 6740 (17.7) | 662 (16.9) | 429 (38.8) | 13855 (36.3) | 1466 (37.4) | 392 (35.4) | 17570 (46) | 1793 (45.7) | 286 (25.8) |
|  | 61 - 80 years | 14385 (20.8) | 1165 (17.7) | 663 (38.8) | 24145 (34.9) | 2514 (38.2) | 607 (35.5) | 30570 (44.2) | 2909 (44.2) | 439 (25.7) |
|  | >80 years | 6410 (37.3) | 328 (29.9) | 188 (56.6) | 4740 (27.6) | 388 (35.4) | 77 (23.2) | 6035 (35.1) | 380 (34.7) | 67 (20.2) |
|  |  |  |  |  |  |  |  |  |  |  |
| Sex, n (row %) | Female | 19595 (20.9) | 1501 (17.3) | 873 (38.1) | 32790 (35) | 3360 (38.7) | 808 (35.3) | 41225 (44) | 3831 (44.1) | 610 (26.6) |
|  | Male | 9300 (24.2) | 770 (21) | 530 (47.7) | 12740 (33.2) | 1306 (35.7) | 331 (29.8) | 16360 (42.6) | 1583 (43.3) | 250 (22.5) |
|  |  |  |  |  |  |  |  |  |  |  |
| Rural Urban classification, n (row %) | Rural | 7490 (22.9) | 990 (25.1) | 1008 (44.5) | 10960 (33.5) | 1395 (35.4) | 652 (28.8) | 14250 (43.6) | 1555 (39.5) | 607 (26.8) |
|  | Urban | 21405 (21.6) | 1281 (15.2) | 395 (34.8) | 34565 (34.8) | 3271 (38.9) | 487 (42.9) | 43335 (43.6) | 3859 (45.9) | 253 (22.3) |
|  |  |  |  |  |  |  |  |  |  |  |
| IMD, n (row %) | 1 (Most deprived) | 5060 (21.6) | 378 (16) | 73 (34) | 8485 (36.2) | 907 (38.3) | 95 (44.2) | 9925 (42.3) | 1084 (45.8) | 47 (21.9) |
|  | 2 | 5785 (22.5) | 455 (16.7) | 187 (35.5) | 8975 (34.9) | 1025 (37.5) | 209 (39.7) | 10930 (42.5) | 1252 (45.8) | 131 (24.9) |
|  | 3 | 6525 (21.9) | 555 (21.4) | 323 (34.1) | 10060 (33.8) | 973 (37.5) | 310 (32.7) | 13160 (44.2) | 1065 (41.1) | 315 (33.2) |
|  | 4 | 5980 (21.6) | 521 (21.9) | 636 (52.7) | 9375 (33.9) | 885 (37.3) | 323 (26.8) | 12325 (44.5) | 968 (40.8) | 247 (20.5) |
|  | 5 (Least deprived) | 5540 (21.8) | 362 (15.9) | 184 (36.4) | 8635 (34) | 876 (38.4) | 202 (39.9) | 11245 (44.2) | 1045 (45.8) | 120 (23.7) |
|  |  |  |  |  |  |  |  |  |  |  |
| Smoking, n (row %) | Never smoked | 11055 (22.3) | 581 (18) | - | 17175 (34.7) | 1234 (38.3) | - | 21290 (43) | 1406 (43.7) | - |
|  | Current smoker | 4120 (22) | 555 (19.6) | - | 6375 (34.1) | 1062 (37.5) | - | 8200 (43.9) | 1217 (42.9) | - |
|  | Ex-smoker | 13635 (21.5) | 1135 (18) | - | 21855 (34.4) | 2370 (37.6) | - | 27970 (44.1) | 2791 (44.3) | - |
|  | Unknown | 85 (25.8) | - | - | 120 (36.4) | - | - | 125 (37.9) | - | - |
|  |  |  |  |  |  |  |  |  |  |  |
| Time since first RA code, mean (standard deviation) |  | 15 (42.9) | 10.52 (7.1) | 7.22 (4.63) | 10 (28.6) | 8.9 (5.34) | 7.74 (5.23) | 10 (28.6) | 9.29 (5.9) | 8 (5.18) |
|  |  |  |  |  |  |  |  |  |  |  |
| BMI, n (row %) | Missing | 4775 (26) | - | - | 5990 (32.6) | - | - | 7620 (41.4) | - | - |
|  | Underweight | 645 (23.7) | - | - | 935 (34.4) | - | - | 1140 (41.9) | - | - |
|  | Healthy range | 7175 (21) | - | - | 12025 (35.2) | - | - | 14995 (43.9) | - | - |
|  | Overweight | 8270 (21.3) | - | - | 13480 (34.8) | - | - | 17035 (43.9) | - | - |
|  | Obese | 6845 (21.3) | - | - | 11125 (34.7) | - | - | 14120 (44) | - | - |
|  | Severe obesity | 1180 (20.3) | - | - | 1975 (33.9) | - | - | 2670 (45.8) | - | - |
|  |  |  |  |  |  |  |  |  |  |  |
| Ethnicity, n (row %) | White | 26125 (21.8) | - | - | 41135 (34.3) | - | - | 52610 (43.9) | - | - |
|  | Asian | 1505 (19.5) | - | - | 2960 (38.3) | - | - | 3270 (42.3) | - | - |
|  | Black | 415 (23.6) | - | - | 595 (33.9) | - | - | 745 (42.5) | - | - |
|  | Mixed | 155 (18.7) | - | - | 325 (39.2) | - | - | 350 (42.2) | - | - |
|  | Other | 220 (22) | - | - | 360 (36) | - | - | 420 (42) | - | - |
|  | Missing | 475 (59) | - | - | 145 (18) | - | - | 185 (23) | - | - |

## Supplementary Table S3: Characteristics of patients by category of difference in the number of rheumatology outpatient appointments in 2021 compared with 2019

|  |  | No appointments either year | | | Fewer appointments | | | Same number or more appointments | | |
| --- | --- | --- | --- | --- | --- | --- | --- | --- | --- | --- |
| Country | | England | Wales | Scotland | England | Wales | Scotland | England | Wales | Scotland |
| Number of individuals | | 26,480 | 2127 | 1348 | 44,650 | 2659 | 1006 | 54,445 | 5203 | 776 |
| Age category, n (row %) | 18 - 40 years | 1320 (17.8) | 110 (14.9) | 124 (50.6) | 2905 (39.2) | 291 (39.3) | 71 (29) | 3180 (39.2) | 339 (45.8) | 50 (20.4) |
|  | 41 - 60 years | 6435 (17.2) | 609 (15.7) | 428 (39.7) | 14040 (37.4) | 1459 (37.7) | 378 (35.1) | 17045 (37.4) | 1805 (46.6) | 272 (25.2) |
|  | 61 - 80 years | 13425 (20.3) | 1126 (17.9) | 632 (39) | 23445 (35.5) | 2408 (38.4) | 572 (35.3) | 29135 (35.5) | 2741 (43.7) | 417 (25.7) |
|  | >80 years | 5300 (36.2) | 282 (31.2) | 164 (57.5) | 4260 (29.1) | 303 (33.6) | 84 (29.5) | 5085 (29.1) | 318 (35.2) | 37 (13) |
|  |  |  |  |  |  |  |  |  |  |  |
| Sex, n (row %) | Female | 17960 (20.1) | 1388 (21.3) | 852 (38.9) | 32205 (36) | 1388 (21.3) | 766 (35) | 39265 (36) | 3746 (57.4) | 573 (26.2) |
|  | Male | 8520 (23.6) | 739 (21.3) | 496 (47.8) | 12440 (34.4) | 1271 (36.7) | 339 (32.7) | 15180 (34.4) | 1457 (42) | 203 (19.6) |
|  |  |  |  |  |  |  |  |  |  |  |
| Rural Urban classification, n (row %) | Rural | 6940 (22.3) | 909 (24.1) | 983 (45.6) | 10680 (34.3) | 1348 (35.8) | 667 (30.9) | 13500 (34.3) | 1508 (40.1) | 508 (23.5) |
|  | Urban | 19545 (20.7) | 1218 (15.2) | 365 (34.1) | 33965 (36) | 3113 (38.8) | 438 (40.9) | 40945 (36) | 3695 (46) | 268 (25) |
|  |  |  |  |  |  |  |  |  |  |  |
| IMD, n (row %) | 1 (Most deprived) | 4610 (20.7) | 366 (16.2) | 63 (31.5) | 8135 (36.6) | 870 (38.5) | 81 (40.5) | 9510 (36.6) | 1022 (45.3) | 56 (28) |
|  | 2 | 5295 (21.7) | 421 (16.1) | 177 (35.8) | 8830 (36.2) | 1005 (38.5) | 186 (37.6) | 10285 (36.2) | 1182 (45.3) | 132 (26.7) |
|  | 3 | 5940 (21) | 525 (21.1) | 303 (34) | 9920 (35) | 944 (38) | 327 (36.7) | 12450 (35) | 1014 (40.8) | 262 (29.4) |
|  | 4 | 5525 (20.9) | 481 (21.1) | 624 (53.9) | 9225 (35) | 821 (36.1) | 325 (28.1) | 11630 (35) | 974 (42.8) | 208 (18) |
|  | 5 (Least deprived) | 5110 (21.1) | 334 (15.4) | 181 (37.3) | 8540 (35.3) | 821 (37.9) | 186 (38.4) | 10570 (35.3) | 1011 (46.7) | 118 (24.3) |
|  |  |  |  |  |  |  |  |  |  |  |
| Smoking, n (row %) | Never smoked | 10275 (21.6) | 566 (18.3) | - | 16835 (35.4) | 1197 (38.6) | - | 20475 (35.4) | 1336 (43.1) | - |
|  | Current smoker | 3750 (21) | 494 (18.1) | - | 6290 (35.3) | 1034 (38) | - | 7775 (35.3) | 1194 (43.9) | - |
|  | Ex-smoker | 12375 (20.7) | 1067 (17.9) | - | 21395 (35.7) | 2230 (37.4) | - | 26085 (35.7) | 2673 (44.8) | - |
|  | Unknown | 85 (26.6) | - | - | 125 (39.1) | - | - | 110 (39.1) | - | - |
|  |  |  |  |  |  |  |  |  |  |  |
| Time since first RA code, mean (standard deviation) |  | 15 (42.9) | 10.55 (7.01) | 7.25 (4.62) | 10 (28.6) | 8.91 (10.40) | 7.84 (5.4) | 10 (28.6) | 9.13 (5.83) | 7.79 (4.93) |
|  |  |  |  |  |  |  |  |  |  |  |
| BMI, n (row %) | Missing | 4320 (24.7) | - | - | 6045 (34.6) | - | - | 7125 (34.6) | - | - |
|  | Underweight | 505 (21.1) | - | - | 880 (36.8) | - | - | 1005 (36.8) | - | - |
|  | Healthy range | 6530 (20.2) | - | - | 11550 (35.7) | - | - | 14230 (35.7) | - | - |
|  | Overweight | 7680 (20.7) | - | - | 13210 (35.6) | - | - | 16180 (35.6) | - | - |
|  | Obese | 6350 (20.7) | - | - | 10970 (35.7) | - | - | 13410 (35.7) | - | - |
|  | Severe obesity | 1100 (19.7) | - | - | 1990 (35.6) | - | - | 2495 (35.6) | - | - |
|  |  |  |  |  |  |  |  |  |  |  |
| Ethnicity, n (row %) | White | 23920 (21) | - | - | 40270 (35.4) | - | - | 49670 (35.4) | - | - |
|  | Asian | 1430 (19) | - | - | 2955 (39.3) | - | - | 3135 (39.3) | - | - |
|  | Black | 370 (21.8) | - | - | 600 (35.4) | - | - | 725 (35.4) | - | - |
|  | Mixed | 140 (17.4) | - | - | 315 (39.1) | - | - | 350 (39.1) | - | - |
|  | Other | 195 (20.4) | - | - | 355 (37.2) | - | - | 405 (37.2) | - | - |
|  | Missing | 425 (57.8) | - | - | 150 (20.4) | - | - | 160 (20.4) | - | - |

## Supplementary Table S4: Logistic regression results measuring characteristics of those who had fewer appointments in 2020 compared to 2019.

|  | | **Univariable, odds ratio (95% confidence intervals)** | | | **Multivariable, odds ratio (95% confidence intervals)** | | | |
| --- | --- | --- | --- | --- | --- | --- | --- | --- |
| **Variable** | **Group** | **England** | **Scotland** | **Wales** | | **England** | **Scotland** | **Wales** |
| Age | 18-40 | 1.04 (0.98, 1.1) | 0.83(0.6, 1.15) | 1.11(0.95, 1.31) | | 1.03 (0.97, 1.09) | 0.62(0.45, 0.85) | 0.94(0.73, 1.2) |
|  | 41-60 | Reference | Reference | Reference | | Reference | Reference | Reference |
|  | 61-80 | 1 (0.97, 1.03) | 0.61(0.45, 0.83) | 1.03(0.95, 1.12) | | 1.01 (0.99, 1.04) | 0.99(0.84, 1.16) | 1.06(0.93, 1.2) |
|  | >80 | 1 (0.95, 1.04) | 0.46(0.34, 0.62) | 0.92(0.8, 1.05) | | 1.02 (0.98, 1.07) | 0.52(0.39, 0.7) | 0.9(0.73, 1.09) |
| Sex | Male | 0.98 (0.95, 1.01) | 0.6(0.44, 0.82) | 0.88(0.81, 0.95) | | 0.99 (0.96, 1.01) | 0.81(0.69, 0.95) | 0.83(0.74, 0.94) |
| Rural-urban classification | Urban | 1.04 (1.01, 1.07) | 0.84(0.61, 1.16) | 1.16(1.07, 1.26) | | 1.02 (0.99, 1.05) | 1.7(1.44, 2) | 1.19(1.04, 1.35) |
| IMD | 1 (Most deprived) | Reference | Reference | Reference | | Reference | Reference | Reference |
|  | 2 | 0.96 (0.92, 1) | 0.55(0.41, 0.73) | 0.97(0.86, 1.08) | | 1 (0.96, 1.05) | 0.91(0.66, 1.26) | 0.93(0.78, 1.1) |
|  | 3 | 0.89 (0.86, 0.93) | 1.06(0.92, 1.22) | 0.97(0.86, 1.09) | | 0.95 (0.91, 0.99) | 0.77(0.56, 1.06) | 1(0.84, 1.19) |
|  | 4 | 0.89 (0.86, 0.93) | 1.86(1.6, 2.16) | 0.96(0.85, 1.08) | | 0.94 (0.9, 0.98) | 0.61(0.44, 0.83) | 0.95(0.79, 1.14) |
|  | 5 (Least deprived) | 0.9 (0.86, 0.94) | 0.78(0.67, 0.91) | 1(0.89, 1.13) | | 0.94 (0.9, 0.98) | 0.88(0.63, 1.22) | 1.08(0.89, 1.29) |
| Ethnicity | White | Reference | Reference | Reference | | Reference | Reference | Reference |
|  | Asian | 1.16 (1.1, 1.22) | - | - | | 1.21 (1.15, 1.28) | - | - |
|  | Black | 1.02 (0.92, 1.14) | - | - | | 1.11 (0.99, 1.24) | - | - |
|  | Mixed | 1.2 (1.03, 1.39) | - | - | | 1.26 (1.08, 1.47) | - | - |
|  | Other | 1.1 (0.95, 1.26) | - | - | | 1.18 (1.03, 1.37) | - | - |
|  | Missing | 1.02 (0.82, 1.27) | - | - | | 1.06 (0.85, 1.32) | - | - |

## Supplementary Table S5: Logistic regression measuring characteristics of those who had fewer appointments in 2021 compared to 2019.

|  | | **Univariable, odds ratio (95% confidence intervals)** | | | **Multivariable, odds ratio (95% confidence intervals)** | | | |
| --- | --- | --- | --- | --- | --- | --- | --- | --- |
| **Variable** | **Group** | **England** | **Scotland** | **Wales** | | **England** | **Scotland** | **Wales** |
| Age | 18-40 | 1.11 (1.05, 1.17) | 0.88(0.63, 1.24) | 1.07(0.91, 1.26) | | 1.1 (1.04, 1.16) | 0.79(0.58, 1.06) | 0.96(0.75, 1.24) |
|  | 41-60 | Reference | Reference | Reference | | Reference | Reference | Reference |
|  | 61-80 | 0.98 (0.95, 1) | 0.85(0.62, 1.17) | 1.03(0.95, 1.12) | | 0.99 (0.96, 1.02) | 0.99(0.84, 1.17) | 1.07(0.94, 1.22) |
|  | >80 | 1.02 (0.97, 1.07) | 0.57(0.42, 0.78) | 0.84(0.72, 0.97) | | 1.04 (0.99, 1.09) | 0.75(0.56, 1) | 0.76(0.61, 0.95) |
| Sex | Male | 1 (0.97, 1.03) | 0.76(0.56, 1.02) | 0.93(0.86, 1.01) | | 1.01 (0.98, 1.04) | 0.93(0.8, 1.1) | 0.86(0.76, 0.97) |
| Rural-urban classification | Urban | 1.05 (1.02, 1.08) | 0.91(0.65, 1.28) | 1.14(1.05, 1.23) | | 1.03 (1, 1.06) | 1.49(1.26, 1.76) | 0.99(0.87, 1.14) |
| IMD | 1 (Most deprived) | Reference | Reference | Reference | | Reference | Reference | Reference |
|  | 2 | 1 (0.96, 1.05) | 0.77(0.58, 1.02) | 1(0.89, 1.12) | | 1.02 (0.98, 1.07) | 0.95(0.68, 1.34) | 0.97(0.81, 1.16) |
|  | 3 | 0.93 (0.9, 0.97) | 1.09(0.95, 1.27) | 0.98(0.87, 1.1) | | 0.97 (0.93, 1.01) | 1.01(0.73, 1.41) | 1.06(0.89, 1.27) |
|  | 4 | 0.93 (0.89, 0.97) | 1.55(1.33, 1.8) | 0.9(0.8, 1.02) | | 0.96 (0.92, 1) | 0.71(0.51, 0.98) | 0.94(0.78, 1.14) |
|  | 5 (Least deprived) | 0.94 (0.91, 0.98) | 0.9(0.77, 1.05) | 0.97(0.86, 1.1) | | 0.97 (0.93, 1.01) | 0.95(0.68, 1.35) | 1(0.82, 1.21) |
| Ethnicity | White | Reference | Reference | Reference | | Reference | Reference | Reference |
|  | Asian | 1.16 (1.1, 1.23) | - | - | | 1.16 (1.1, 1.22) | - | - |
|  | Black | 1.02 (0.92, 1.14) | - | - | | 1.05 (0.94, 1.17) | - | - |
|  | Mixed | 1.12 (0.96, 1.3) | - | - | | 1.12 (0.96, 1.31) | - | - |
|  | Other | 1.08 (0.93, 1.24) | - | - | | 1.1 (0.95, 1.27) | - | - |
|  | Missing | 1.11 (1.05, 1.17) | 0.88(0.63, 1.24) | 1.07(0.91, 1.26) | | 1.17 (0.93, 1.46) | - | - |

##

## Supplementary Table S6: OpenSAFELY Collaborative

| **First name and middle initial** | **Surname** |
| --- | --- |
| Alex J | Walker |
| Brian | MacKenna |
| Peter | Inglesby |
| Ben | Goldacre |
| Helen J | Curtis |
| Caroline E | Morton |
| Jessica | Morley |
| Amir | Mehrkar |
| Sebastian CJ | Bacon |
| George | Hickman |
| Richard | Croker |
| David | Evans |
| Tom | Ward |
| Nicholas J | DeVito |
| Louis | Fisher |
| Amelia CA | Green |
| Jon | Massey |
| Rebecca M | Smith |
| William J | Hulme |
| Simon | Davy |
| Colm D | Andrews |
| Lisa EM | Hopcroft |
| Henry | Drysdale |
| Iain | Dillingham |
| Robin Y | Park |
| Rose | Higgins |
| Christine | Cunningham |
| Milan | Wiedemann |
| Linda | Nab |
| Steven | Maude |
| Orla | Macdonald |
| Ben FC | Butler-Cole |
| Thomas | O'Dwyer |
| Catherine L | Stables |
| Christopher | Wood |
| Andrew D | Brown |
| Victoria | Speed |
| Lucy | Bridges |
| Andrea L | Schaffer |
| Caroline E | Walters |
| Christopher T | Rentsch |
| Krishnan | Bhaskaran |
| Anna | Schultze |
| Elizabeth J | Williamson |
| Helen I | McDonald |
| Laurie A | Tomlinson |
| Rohini | Mathur |
| Rosalind M | Eggo |
| Kevin | Wing |
| Angel YS | Wong |
| John | Tazare |
| Richard | Grieve |
| Daniel J | Grint |
| Sinead | Langan |
| Kathryn E | Mansfield |
| Ian J | Douglas |
| Stephen JW | Evans |
| Liam | Smeeth |
| Jemma L | Walker |
| Viyaasan | Mahalingasivam |
| Harriet | Forbes |
| Thomas E | Cowling |
| Emily L | Herrett |
| Ruth E | Costello |
| Bang | Zheng |
| Edward P K | Parker |
| Christopher | Bates |
| Jonathan | Cockburn |
| John | Parry |
| Frank | Hester |
| Sam | Harper |
| Shaun | O'Hanlon |
| Alex | Eavis |
| Richard | Jarvis |
| Dima | Avramov |
| Paul | Griffiths |
| Aaron | Fowles |
| Nasreen | Parkes |
| Brian | Nicholson |
| Rafael | Perera |
| David | Harrison |
| Kamlesh | Khunti |
| Jonathan AC | Sterne |
| Jennifer | Quint |

## Supplementary Table S7: The LH&W NCS Collaborative

| **First name** | **Surname** |
| --- | --- |
| Nishi | Chaturvedi |
| Chloe | Park |
| Alisia | Carnemolla |
| Dylan | Williams |
| Anika | Knueppel |
| Andy | Boyd |
| Emma L | Turner |
| Katharine M | Evans |
| Richard | Thomas |
| Samantha | Berman |
| Stela | McLachlan |
| Matthew | Crane |
| Rebecca | Whitehorn |
| Jacqui | Oakley |
| Diane | Foster |
| Hannah | Woodward |
| Kirsteen C | Campbell |
| Nicholas | Timpson |
| Alex | Kwong |
| Ana Goncalves | Soares |
| Gareth | Griffith |
| Renin | Toms |
| Louise | Jones |
| Herbert, | Annie |
| Ruth | Mitchell |
| Tom | Palmer |
| Jonathan | Sterne |
| Venexia | Walker |
| Lizzie | Huntley |
| Laura | Fox |
| Rachel | Denholm |
| Rochelle | Knight |
| Kate | Northstone |
| Arun | Kanagaratnam |
| Elsie | Horne |
| Harriet | Forbes |
| Teri | North |
| Kurt | Taylor |
| Marwa AL | Arab |
| Scott | Walker |
| Jose IC | Coronado |
| Arun S | Karthikeyan |
| George | Ploubidis |
| Bettina | Moltrecht |
| Charlotte | Booth |
| Sam | Parsons |
| Bozena | Wielgoszewska |
| Charis | Bridger-Staatz |
| Claire | Steves |
| Ellen | Thompson |
| Paz | Garcia |
| Nathan | Cheetham |
| Ruth | Bowyer |
| Maxim | Freydin |
| Amy | Roberts |
| Ben | Goldacre |
| Alex | Walker |
| Jess | Morley |
| William | Hulme |
| Linda | Nab |
| Louis | Fisher |
| Brian | MacKenna |
| Colm | Andrews |
| Helen | Curtis |
| Lisa | Hopcroft |
| Amelia | Green |
| Praveetha | Patalay |
| Jane | Maddock |
| Kishan | Patel |
| Jean | Stafford |
| Wels | Jacques |
| Kate | Tilling |
| John | Macleod |
| Eoin | McElroy |
| Anoop | Shah |
| Richard | Silverwood |
| Spiros | Denaxas |
| Robin | Flaig |
| Daniel | McCartney |
| Archie | Campbell |
| Laurie | Tomlinson |
| John | Tazare |
| Bang | Zheng |
| Liam | Smeeth |
| Emily | Herrett |
| Thomas | Cowling |
| Kate | Mansfield |
| Ruth E | Costello |
| Kevin | Wang |
| Kathryn | Mansfield |
| Viyaasan | Mahalingasivam |
| Ian | Douglas |
| Sinead | Langan |
| Sinead | Brophy |
| Michael | Parker |
| Jonathan | Kennedy |
| Rosie | McEachan |
| John | Wright |
| Kathryn | Willan |
| Ellena | Badrick |
| Gillian | Santorelli |
| Tiffany | Yang |
| Bo | Hou |
| Andrew | Steptoe |
| Di Gessa, | Giorgio |
| Jingmin | Zhu |
| Paola | Zaninotto |
| Angela | Wood |
| Genevieve | Cezard |
| Samantha | Ip |
| Tom | Bolton |
| Alexia | Sampri |
| Elena | Rafeti |
| Fatima | Almaghrabi |
| Aziz | Sheikh |
| Syed A | Shah |
| Vittal | Katikireddi |
| Richard | Shaw |
| Olivia | Hamilton |
| Michael | Green |
| Theocharis | Kromydas |
| Daniel | Kopasker |
| Felix | Greaves |
| Robert | Willans |
| Fiona | Glen |
| Steve | Sharp |
| Alun | Hughes |
| Andrew | Wong |
| Lee Hamill | Howes |
| Alicja | Rapala |
| Lidia | Nigrelli |
| Fintan | McArdle |
| Chelsea | Beckford |
| Betty | Raman |
| Richard | Dobson |
| Amos | Folarin |
| Callum | Stewart |
| Yatharth | Ranjan |
| Jd | Carpentieri |
| Laura | Sheard |
| Chao | Fang |
| Sarah | Baz |
| Andy | Gibson |
| John | Kellas |
| Stefan | Neubauer |
| Stefan | Piechnik |
| Elena | Lukaschuk |
| Laura C | Saunders |
| James M | Wild |
| Stephen | Smith |
| Peter | Jezzard |
| Elizabeth | Tunnicliffe |
| Zeena-Britt | Sanders |
| Lucy | Finnigan |
| Vanessa | Ferreira |
| Mark | Green |
| Rebecca | Rhead |
| Milla | Kibble |
| Yinghui | Wei |
| Agnieszka | Lemanska |
| Francisco | Perez-Reche |
| Dominik | Piehlmaier |
| Lucy | Teece |
| Edward | Parker |

## Supplementary text S8: RHEUMAPS study investigators

The RHEUMAPS study investigators include: Rosemary J Hollick (Chief Investigator) (Senior Clinical Lecturer, University of Aberdeen, Honorary Consultant Rheumatologist, NHS Grampian), Corri Black (Emeritus Professor, University of Aberdeen), Sinead Brophy (Professor of Health Data Science, Swansea University), Ernest Choy (Head of Rheumatology and Translational Research, Cardiff University), Gary Macfarlane (Clinical Chair in Epidemiology, University of Aberdeen), Louise Bennett (University of Glasgow), Lorna Philip (Professor of Geography and Environment, University of Aberdeen), Michelle Stevenson (Patient Partner), Denise McFarlane (GP, NHS Grampian and Chair, External Advisory Group), Laura Moir (Study Coordinator, University of Aberdeen) and Public Contributors Ian Allotay, Philip Bell, Amanda Cheesley, Charlotte Marlow, Farzana Kausir, Emily Lam and Inga Wood.

## Supplementary Table S9: RECORD checklist

The RECORD statement (RECORD) checklist of items, extended from the STROBE

| Item No | STROBE items | RECORD items | Page No |
| --- | --- | --- | --- |
| 1 | (a) Indicate the study’s design with a commonly used term in the title or the abstract.  (b) Provide in the abstract an informative and balanced summary of what was done and what was found. | 1.1: The type of data used should be specified in the title or abstract. When possible, the name of the databases used should be included.  1.2: If applicable, the geographical region and timeframe within which the study took place should be reported in the title or abstract.  1.3: If linkage between databases was conducted for the study, this should be clearly stated in the title or abstract. | 1-2 |
| 2 | Explain the scientific background and rationale for the investigation being reported. | — | 3 |
| 3 | State specific objectives, including any prespecified hypotheses. | — | 3 |
| 4 | Present key elements of study design early in the paper. | — | 3-5 |
| 5 | Describe the setting, locations, and relevant dates, including periods of recruitment, exposure, follow-up, and data collection. | — | 3-4 |
| 6 | (a) Cohort study—give the eligibility criteria, and the sources and methods of selection of participants. Describe methods of follow-up. Case-control study—give the eligibility criteria, and the sources and methods of case ascertainment and control selection. Give the rationale for the choice of cases and controls. Cross sectional study—give the eligibility criteria, and the sources and methods of selection of participants.  (b) Cohort study—for matched studies, give matching criteria and number of exposed and unexposed. Case-control study—for matched studies, give matching criteria and the number of controls per case. | 6.1: The methods of study population selection (such as codes or algorithms used to identify participants) should be listed in detail. If this is not possible, an explanation should be provided.  6.2: Any validation studies of the codes or algorithms used to select the population should be referenced. If validation was conducted for this study and not published elsewhere, detailed methods and results should be provided.  6.3: If the study involved linkage of databases, consider use of a flow diagram or other graphical display to demonstrate the data linkage process, including the number of individuals with linked data at each stage. | 4 |
| 7 | Clearly define all outcomes, exposures, predictors, potential confounders, and effect modifiers. Give diagnostic criteria, if applicable. | 7.1: A complete list of codes and algorithms used to classify exposures, outcomes, confounders, and effect modifiers should be provided. If these cannot be reported, an explanation should be provided. | 4-5 |
| 8 | For each variable of interest, give sources of data and details of methods of assessment (measurement). Describe comparability of assessment methods if there is more than one group. | — | 4-5 |
| 9 | Describe any efforts to address potential sources of bias. | — | 4-5 |
| 10 | Explain how the study size was arrived at. | — | 6 |
| 11 | Explain how quantitative variables were handled in the analyses. If applicable, describe which groupings were chosen, and why. | — | 4-5 |
| 12 | (a) Describe all statistical methods, including those used to control for confounding.  (b) Describe any methods used to examine subgroups and interactions.  (c) Explain how missing data were addressed.  (d) Cohort study—if applicable, explain how loss to follow-up was addressed. Case-control study—if applicable, explain how matching of cases and controls was addressed. Cross sectional study—if applicable, describe analytical methods taking account of sampling strategy.  (e) Describe any sensitivity analyses. | — | 5-6 |
| 12 | — | 12.1: Authors should describe the extent to which the investigators had access to the database population used to create the study population.  12.2: Authors should provide information on the data cleaning methods used in the study. | 4-6 |
| 12 | — | 12.3: State whether the study included person level, institutional level, or other data linkage across two or more databases. The methods of linkage and methods of linkage quality evaluation should be provided. | 3-6 |
| 13 | (a) Report the numbers of individuals at each stage of the study (eg, numbers potentially eligible, examined for eligibility, confirmed eligible, included in the study, completing follow-up, and analysed).  (b) Give reasons for non-participation at each stage.  (c) Consider use of a flow diagram. | 13.1: Describe in detail the selection of the individuals included in the study (that is, study population selection) including filtering based on data quality, data availability, and linkage. The selection of included individuals can be described in the text or by means of the study flow diagram. | 6 |
| 14 | (a) Give characteristics of study participants (eg, demographic, clinical, social) and information on exposures and potential confounders.  (b) Indicate the number of participants with missing data for each variable of interest.  (c) Cohort study—summarise follow-up time (eg, average and total amount). | — | 6 |
| 15 | Cohort study—report numbers of outcome events or summary measures over time. Case-control study—report numbers in each exposure category, or summary measures of exposure. Cross sectional study—report numbers of outcome events or summary measures. | — | 6-8 |
| 16 | (a) Give unadjusted estimates and, if applicable, confounder adjusted estimates and their precision (eg, 95% confidence intervals). Make clear which confounders were adjusted for and why they were included.  (b) Report category boundaries when continuous variables are categorised.  (c) If relevant, consider translating estimates of relative risk into absolute risk for a meaningful time period. | — | 7-8 |
| 17 | Report other analyses done—eg, analyses of subgroups and interactions, and sensitivity analyses. | — | N/A |
| 18 | Summarise key results with reference to study objectives. | — | 8 |
| 19 | Discuss limitations of the study, taking into account sources of potential bias or imprecision. Discuss both direction and magnitude of any potential bias. | 19.1: Discuss the implications of using data that were not created or collected to answer the specific research question(s). Include discussion of misclassification bias, unmeasured confounding, missing data, and changing eligibility over time, as they pertain to the study being reported. | 8-9 |
| 20 | Give a cautious overall interpretation of results considering objectives, limitations, multiplicity of analyses, results from similar studies, and other relevant evidence. | — | 8-12 |
| 21 | Discuss the generalisability (external validity) of the study results. | — | 8 |
| 22 | Give the source of funding and the role of the funders for the present study and, if applicable, for the original study on which the present article is based. | — | 12-13 |
| 22 | — | 22.1: Authors should provide information on how to access any supplemental information such as the study protocol, raw data, or programming code. | 6 |

RECORD=reporting of studies conducted using observational routinely collected data; STROBE=strengthening the reporting of observational studies in epidemiology.
